# Low-Thermal-Conductivity (*M*S)_1+*x*_(TiS_2_)_2_ (*M* = Pb, Bi, Sn) Misfit Layer Compounds for Bulk Thermoelectric Materials

**DOI:** 10.3390/ma3042606

**Published:** 2010-04-06

**Authors:** Chunlei Wan, Yifeng Wang, Ning Wang, Kunihito Koumoto

**Affiliations:** 1 Graduate School of Engineering, Nagoya University, Nagoya 464-8603, Japan; E-Mails: chunlei.wan@gmail.com (C.W.); yifeng.wang@apchem.nagoya-u.ac.jp (Y.W.); ning.wang@apchem.nagoya-u.ac.jp (N.W.); 2 CREST, Japan Science and Technology Agency, Tokyo 101-0075, Japan

**Keywords:** thermoelectric materials, misfit layer compounds, sound velocity, interlayer bonding

## Abstract

A series of (*M*S)_1+*x*_(TiS_2_)_2_ (*M* = Pb, Bi, Sn) misfit layer compounds are proposed as bulk thermoelectric materials. They are composed of alternating rock-salt-type *M*S layers and paired trigonal anti-prismatic TiS_2_ layers with a van der Waals gap. This naturally modulated structure shows low lattice thermal conductivity close to or even lower than the predicted minimum thermal conductivity. Measurement of sound velocities shows that the ultra-low thermal conductivity partially originates from the softening of the transverse modes of lattice wave due to weak interlayer bonding. Combined with a high power factor, the misfit layer compounds show a relatively high *ZT* value of 0.28~0.37 at 700 K.

## 1. Introduction

Thermoelectric materials have been considered as an effective solution for the increasing energy crisis nowadays [1]. By taking advantage of the Seebeck effect, thermoelectric materials can generate electricity from waste heat that widely exists in automobile exhaust and various industrial processes. The figure of merit of thermoelectric materials is defined as follows: *ZT* = *S*^2^*σ*/*k,* where *S*, *σ*, and *k* represent Seebeck coefficient, electrical conductivity and thermal conductivity, respectively. Since the *ZT* value of the current materials is too low for cost-effective applications, various efforts have been made to improve it. The concept of phonon-glass electron-crystal (PGEC) was proposed and has become a general guideline for developing new thermoelectric materials [2]. In order to obtain the PGEC materials, the idea of complex structure was put forward which imagines a material with distinct regions providing different functions [1]. It is believed that the ideal thermoelectric material would have regions of the structure composed of a high-mobility semiconductor that provides the electron-crystal electronic structure, interwoven with a phonon-glass. The phonon-glass region would be ideal for housing dopants and disordered structures without disrupting the carrier mobility in the electron-crystal region [1].

Based on the above ideas, a series of misfit layer compounds of composition (*M*S)_1+x_(TiS_2_)_2_ (*M* = Pb, Bi, Sn) are investigated here. They consist of an alternating stacking of CdI_2_-type TiS_2_ trigonal anti-prismatic layers and rock-salt-type *M*S slabs, which could be viewed as a natural superlattice [3]. The TiS_2_ layer can provide thermopower as well as electron pathway, according to Imai’s research on TiS_2_ single crystals [4]. The *M*S layer was intercalated into the gap of the TiS_2_ to form a modulated structure which would suppress the transport of phonons by the interaction between the *M*S layer and TiS_2_ layer and/or disruption of the periodicity of TiS_2_ in the direction perpendicular to the layers. The structure and physical properties of misfit layer compounds have been intensively investigated in the 1990s [3,5,6]. In the present study, the thermoelectric performance of these compounds was examined.

## 2. Results and Discussion

### 2.1. Crystal structure and XRD patterns

**Figure 1:**
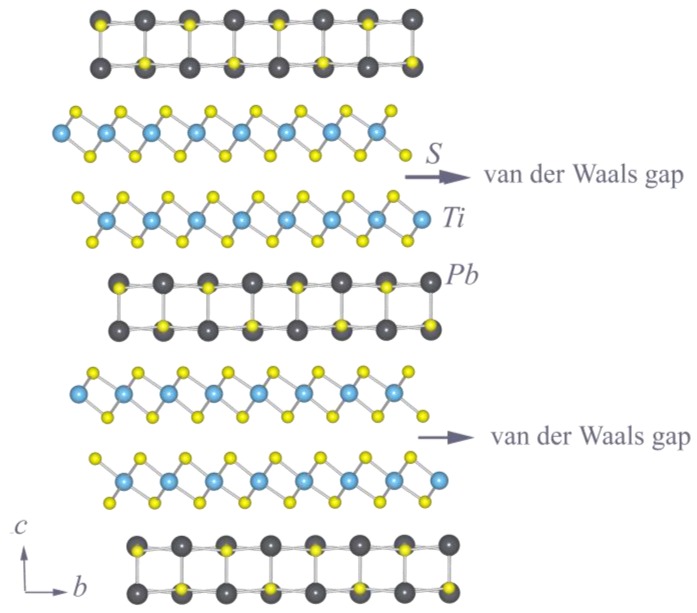
Crystal structure of (PbS)_1.18_(TiS_2_)_2_ along the incommensurate direction.

Generally, the crystal structure of (*M*S)_1+*x*_(TiS_2_)_2_ is composed of a layer of *M*S sandwiched between two TiS_2_ layers with a van der Waals gap [3]. The crystal structure of (PbS)_1.18_(TiS_2_)_2_ has been refined from XRD data [7] and is shown in Figure 1. The Pb and S(1) atom of the PbS subsystem are in 4(*i*) sites of space group C2/m; each Pb atom is coordinated by five S atoms located at the corners of a slightly distorted square pyramid (NaC1 structure type). As Pb atoms protrude from the sulfur planes on both sides, each Pb atom is also bonded to two or three S atoms of the TiS_2_ slabs by weak covalent force. The atoms of the (TiS_2_)_2_ subsystem are on 2(*e*) sites of space group C2_1_/m. Each Ti atom is coordinated by six S atoms in a trigonal antiprismatic arrangement. The (TiS_2_)_2_ slab is slightly distorted compared with 1*T*-TiS_2 ,_in which Ti is octahedrally coordinated. It is seen that the stacking of the two adjacent TiS_2_ sandwiches are the same as in l*T*-TiS_2_.

**Figure 2:**
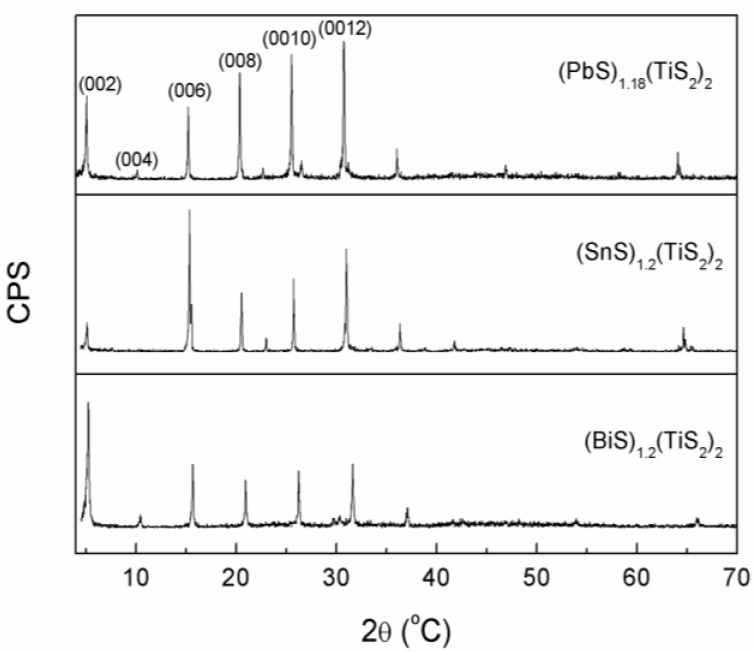
XRD patterns of (BiS)_1.2_(TiS_2_)_2_, (SnS)_1.2_(TiS_2_)_2_ and (PbS)_1.18_(TiS_2_)_2_.

The XRD patterns of the surfaces of the (*M*S)_1+*x*_(TiS_2_)_2_ sintered bodies perpendicular to the pressing direction are shown in Figure 2. Sharp (0 0 *l*) peaks which correspond to the planes perpendicular to the *c*-axis can be observed and very few (*h* *k* *l*) planes are detected, showing that the *c*-axes are preferentially oriented along the pressing direction. The atomic bondings in these misfit layer compounds are highly anisotropic, and the atomic bondings within the layers must be strong due to high covalency and the interlayer bonding formed mainly by van der Waals force is very weak. Under the pressure during SPS sintering, the crystals tend to slide along the layers and deflect until the layers become aligned perpendicular to the pressure, thereby resulting in high preferred-orientation of the (0 0 *l*) planes. Rocking curve is also measured to characterize the degree of preferred orientation. It shows that the full width at half maximum (FWHM) of the (0 0 12) peak of (BiS)_1.18_(TiS_2_)_2_, (SnS)_1.2_(TiS_2_)_2_ and (PbS)_1.18_(TiS_2_)_2_ are 17.6°, 15.1° and 17.2° respectively. Although the values are not as low as those of the films, the degree of preferred orientation for the (0 0 *l*) planes of these polycrystalline samples is high enough to approach the in-plane transport prosperities of a single crystal. There are some minor peaks between 20°-30° in (PbS)_1.18_(TiS_2_)_2_ and (SnS)_1.2_(TiS_2_)_2_, which may have arisen from the stage-1 compounds (PbS)_1.18_TiS_2_ and (SnS)_1.2_TiS_2_, but their presence should have little influence on the thermoelectric properties because their content is negligible.

### 2.2. Electrical properties

**Figure 3:**
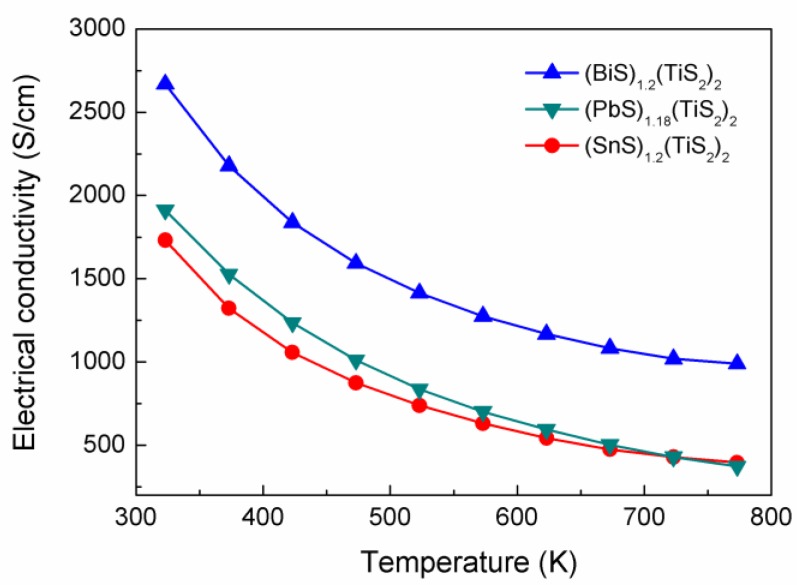
Electrical conductivities of (BiS)_1.2_(TiS_2_)_2_, (SnS)_1.2_(TiS_2_)_2_ and (PbS)_1.18_(TiS_2_)_2_.

As shown in Figure 3, all the (BiS)_1.2_(TiS_2_)_2_, (SnS)_1.2_(TiS_2_)_2_ and (PbS)_1.18_(TiS_2_)_2_ compounds show metallic electrical conductivities of the order of 1,700~2,700 S/cm at room temperature. The electrical conductivity decreases in the sequence of Bi, Pb, Sn for (*M*S)_1+*x*_(TiS_2_)_2_ over the whole temperature range.

**Figure 4:**
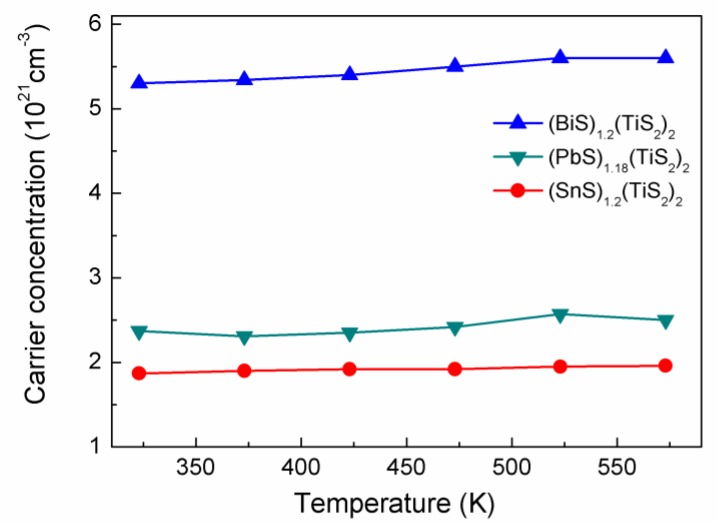
Carrier concentrations of (BiS)_1.2_(TiS_2_)_2_, (SnS)_1.2_(TiS_2_)_2_ and (PbS)_1.18_(TiS_2_)_2_.

The electrical conductivity of materials is determined by the carrier concentration and mobility. Hall measurement was performed to analyze the electron transport properties in these misfit layer compounds. The Hall coefficients are all negative, showing that the dominant carriers in these compounds are electrons. As shown in Figure 4, all the compositions show high carrier concentrations which are almost temperature independent, supporting the metallic conduction mechanism. For the (*M*S)_1+*x*_(TiS_2_)_2_ compositions, the carrier concentration decreases in the sequence of Bi, Sn, Pb which is consistent with the tendency of electrical conductivity. It has been known that pure TiS_2_ is a small-bandgap semiconductor and the carrier concentration is 2.8 × 10^20^ cm^-3^ at room temperature [4]. Since the misfit layer compound can be viewed as composite lattice of the *M*S layer and the TiS_2_ layer, the large carrier concentrations of the misfit layer compounds are believed to originate from electron transfer from the *M*S layer to the TiS_2_ layer [3]. From the carrier concentrations and the lattice parameters, we can estimate the number of electrons per Ti atom received for (BiS)_1.2_(TiS_2_)_2_, (SnS)_1.2_(TiS_2_)_2_ and (PbS)_1.18_(TiS_2_)_2_ is 0.45, 0.16 and 0.2, respectively. Much more electron transfer takes place in (BiS)_1.2_(TiS_2_)_2_ than the other two compositions, because the valence of bismuth is 3+ here and one can easily deduce that one electron can be transferred from one BiS layer to two TiS_2_ layers, leading to that each Ti atom receive 0.6 electrons, which is in reasonable agreement with the above estimation.

**Figure 5:**
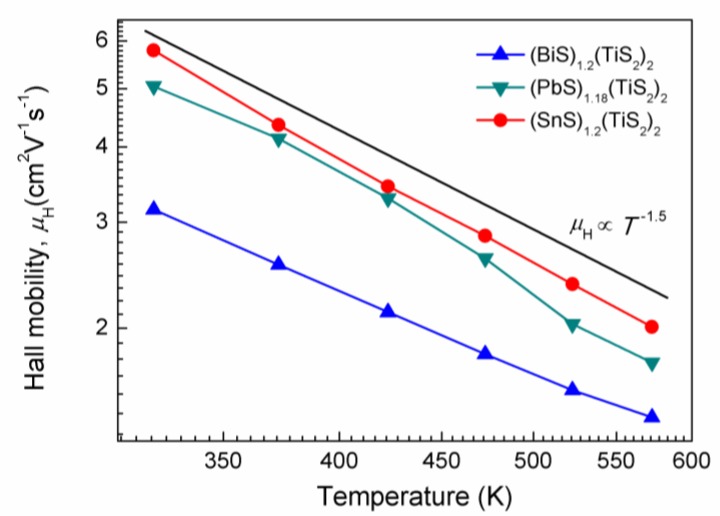
Hall mobilities for (BiS)_1.2_(TiS_2_)_2_, (SnS)_1.2_(TiS_2_)_2_ and (PbS)_1.18_(TiS_2_)_2_.

The Hall mobilities for the (*M*S)_1+*x*_(TiS_2_)_2_ compositions are plotted in Figure 5. The mobilities for all the compositions have temperature dependences proportional to *T* ^-1.5^, showing that the electrons are mainly scattered by acoustic phonons. The degree of orientation of the (0 0 *l*) planes in these polycrystalline samples may affect the mobility, as the electron mobility is much lower in the cross-plane direction of these misfit layer compounds [8]. However, the similar FWHM of the rocking curve shows that the degree of orientation is close for these three compositions and its effect on the mobility is limited.

The mobility in the (*M*S)_1+*x*_(TiS_2_)_2_ decreases in the order of Sn > Pb > Bi which is almost opposite to that of carrier concentration. The electron transfer from the *M*S layers to the TiS_2_ layers may also change the effective mass, resulting in different mobilities. An estimation of the effective mass will be shown below.

**Figure 6:**
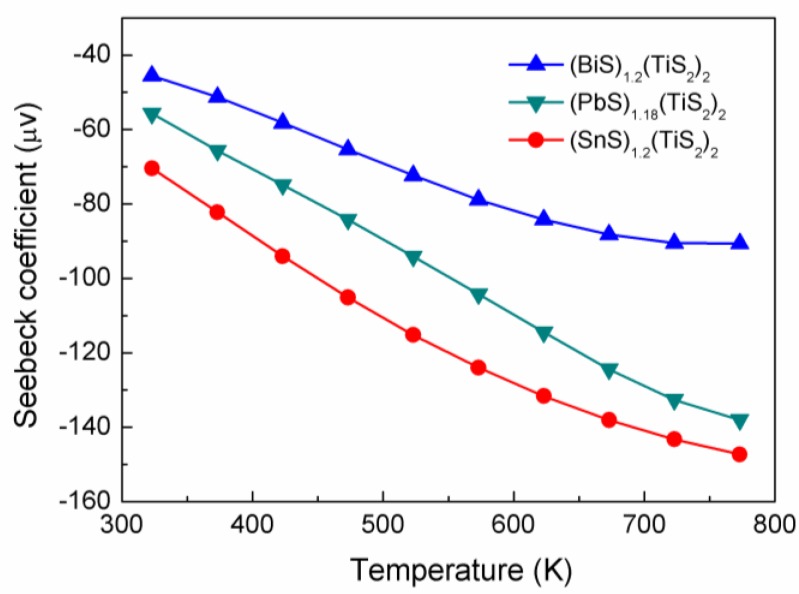
Seebeck coefficients of (BiS)_1.2_(TiS_2_)_2_, (SnS)_1.2_(TiS_2_)_2_ and (PbS)_1.18_(TiS_2_)_2_.

The (*M*S)_1+*x*_(TiS_2_)_2_ compositions show a relatively large Seebeck coefficient, as shown in Figure 6. It can also be seen that the absolute Seebeck coefficient decreases in the order of Sn>Pb>Bi. In these intercalation compounds, electrical properties can be described by a rigid band model, which means that the only change in the electronic structure of the host is a change in a degree of band filling due to electron donation from the intercalated species to the host [9]. It is realized that the *d* orbitals of Ti plays an important role in determining the physical properties of TiS_2_-based materials and the degree of band filling, their energy levels and the width of the *d*-band significantly affect their thermoelectric properties [9]. Accordingly, the Seebeck coefficient decreasing in the order of Sn>Pb>Bi strongly suggests an increase in the number of electrons per Ti atom received, namely indicating higher degree of band filling was achieved.

The density-of-states (DOS) effective mass, m^*^, one of the main factors determining *S*, were estimated by the use of the following equations [10]:

(1) $$m*=\frac{h^{2}}{2k_{B}T}{\left[\frac{n_{e}}{4\pi F_{1/2}(\xi )}\right]}^{2/3}$$

where *h*, *k_B_*, *n_e_*, *F_n_* and *ξ* are the Plank constant, the Boltzmann constant, the carrier concentration, the Fermi integral, and the chemical potential, respectively. *F_n_*(*ξ*) and *S* can be expressed as [10]:

(2) $$F_{n}\left(\xi \right)=\int_{0}^{\infty }\frac{x^{n}}{1+e^{x-\xi }}dx$$

(3) $$S=-\frac{k_{B}}{e}\left[\frac{\left(r+2\right)F_{r+1}\left(\xi \right)}{\left(r+1\right)F_{r}\left(\xi \right)}-\xi \right]$$

where *e* is the electron charge, and *r* is the carrier scattering parameter of relaxation time which was assumed to be *r* = 0 since the carriers are scattered only by acoustic phonons. The m* values for (BiS)_1.2_(TiS_2_)_2_, (SnS)_1.2_(TiS_2_)_2_ and (PbS)_1.18_(TiS_2_)_2_ were calculated to be 6.3 *m*_0_, 4.8 *m*_0_ and 4.5*m*_0_, respectively, where *m*_0_ is the bare electron mass. It can be seen that (BiS)_1.2_(TiS_2_)_2_ has the highest effective mass, resulting in the lowest mobility as shown in Figure 5.

**Figure 7:**
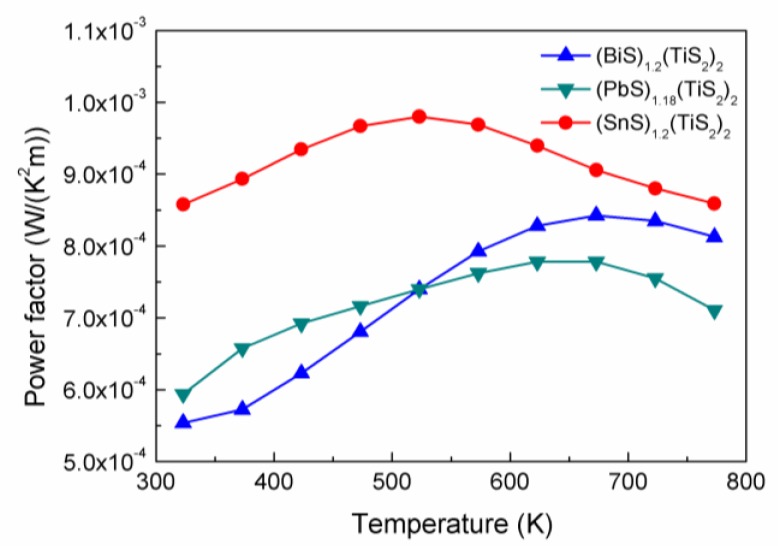
Power factors of (BiS)_1.2_(TiS_2_)_2_, (SnS)_1.2_(TiS_2_)_2_ and (PbS)_1.18_(TiS_2_)_2_.

As shown in Figure 7, the power factors of the (*M*S)_1+*x*_(TiS_2_)_2_ compositions fall within the range of 5 × 10^-4^ to 10^-3^ W/K^2^m, which is much lower than the conventional thermoelectric material Bi_2_Te_3_ (~5 × 10^-3^ W/K^2^m). At lower temperatures, the power factors almost increase in the order of Bi < Pb < Sn, indicative of increased carrier concentration. Although the carrier concentration in (*M*S)_1+*x*_(TiS_2_)_2_ is not yet optimized, it can be expected that further reduction in carrier concentration would increase the power factor. Acceptor doping may be employed to reduce the carrier concentration as in the case of TiS_2_ doped with Mg and Cd [11,12].

### 2.3. Thermal conductivity

**Figure 8:**
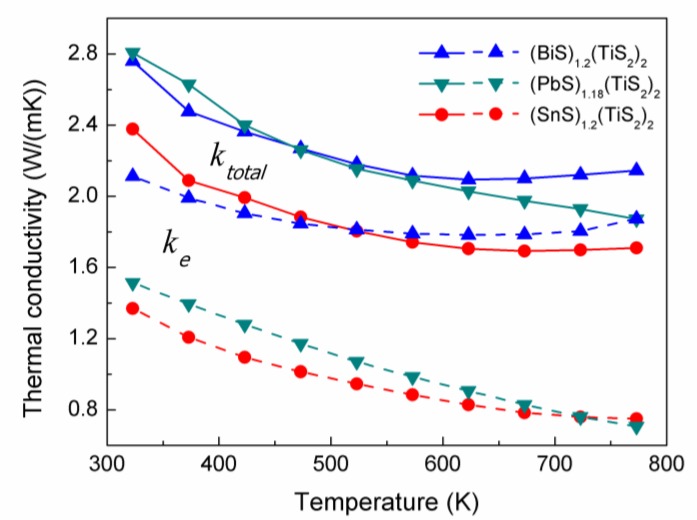
Total thermal conductivities (*k_total_*, solid line) and electron thermal conductivities (*k_e_*, dashed line) of (BiS)_1.2_(TiS_2_)_2_, (SnS)_1.2_(TiS_2_)_2_ and (PbS)_1.18_(TiS_2_)_2_.

As shown in Figure 8, the (*M*S)_1+*x*_(TiS_2_)_2_ compositions exhibit relatively low thermal conductivity. (SnS)_1.2_(TiS_2_)_2_ has lower thermal conductivity than the other compositions in the whole temperature range. Since the thermal conductivity comes from two sources: (1) electrons and holes transporting heat (*k_e_*) and (2) phonons travelling through the lattice (*k_l_*), the electronic thermal conductivity (*k*_e_) is directly related to the electrical conductivity through the Wiedemann-Franz law: *k*_e_=*L*_0_*Tσ*, where the Lorenz number *L*_0_ is 2.44 × 10^-8^ J^-2^C^-2^K^-2^. The values of *k_e_* of these (*M*S)_1+*x*_(TiS_2_)_2_ compositions were calculated and plotted in Figure 8. It can be seen that *k*_e_ largely contributes to the total thermal conductivity, especially in (BiS)_1.2_(TiS_2_)_2_. (SnS)_1.2_(TiS_2_)_2_ has the lowest carrier concentration and electrical conductivity, resulting in the lowest *k*_e_ and also the lowest *k_total_*.

**Figure 9:**
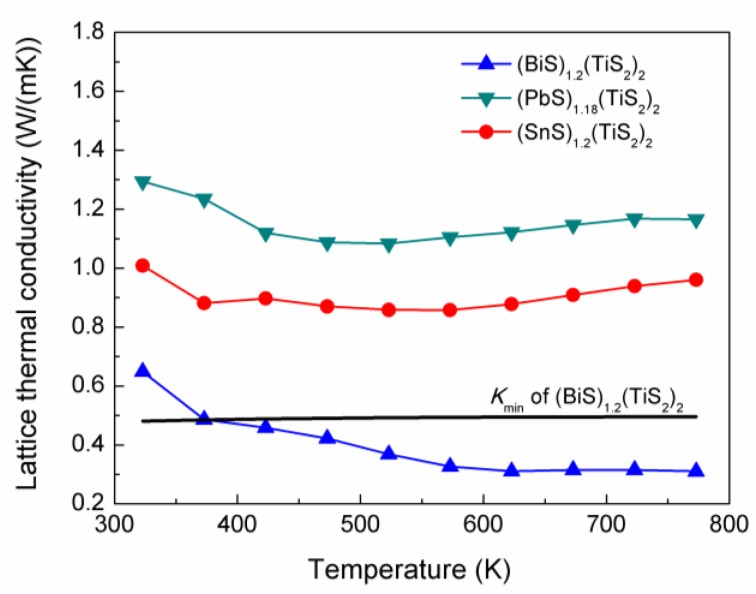
Lattice thermal conductivities of (BiS)_1.2_(TiS_2_)_2_, (SnS)_1.2_(TiS_2_)_2_ and (PbS)_1.18_(TiS_2_)_2_. The calculated minimum thermal conductivity for (BiS)_1.2_(TiS_2_)_2_ is also included.

The lattice thermal conductivity was then calculated by subtracting *k_e_* from *k_total_*, which is shown in Figure 9. It can be noticed that (*M*S)_1+*x*_(TiS_2_)_2_ has extremely low lattice thermal conductivity, which can be related to their modulated structure. (BiS)_1.2_(TiS_2_)_2_ exhibits the lowest *k_l_* and can even reach 0.3 W/mK around 700K. The minimum thermal conductivity can be calculated for this composition from the equation [13]:

(4) $$k_{\min}={\left(\frac{\pi }{6}\right)}^{1/3}k_{B}n^{2/3}\sum_{i}v_{i}{\left(\frac{T}{\theta }\right)}^{2}\int_{0}^{\theta_{i}/T}\frac{x^{3}e^{x}}{{\left(e^{x}-1\right)}^{2}}dx$$

The sum is taken over the three sound modes including two transverse and one longitudinal modes with the speed of sound *v*_i_. *θ*_i_ is the Debye temperature for each polarization, *θ_i_* *= υ_i_(ħ/k_B_)6π^2^n)*^⅓^, where *n* is the number density of atoms [13]. Using the measured values of *V*_L_, *V*_T1_, *V*_T2_, the *k*_min_ was calcuated and shown in Figure 9. *k*_l_ of (BiS)_1.2_(TiS_2_)_2_ is even lower than *k*_min_, which can hardly be observed in the bulk materials.

**Figure 10:**
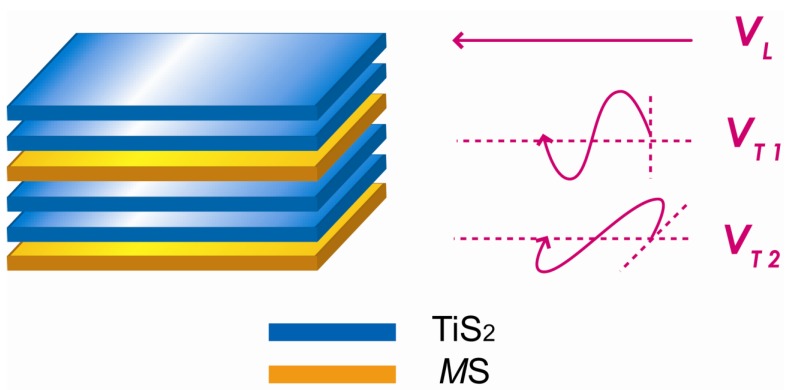
Schematic illustration of the longitudinal and transverse sound velocities of the layered (*M*S)_1+x_(TiS_2_)_2_ compounds.

To analyze the ultra-low thermal conductivity, the kinetic theory of thermal conductivity was used:

*k* = 1/3*C*_v_*lV* (5)

where *C*_v_, *l* and *V* represent the heat capacity, phonon mean free path and speed of sound, respectively. The heat capacity makes limited contribution to the low thermal conductivity, as the heat capacity approaches 3*k*_B_ per atom at temperatures higher than the Debye temperature, according to the Dulong-Petit law. The phonon mean free path is restricted by various phonon scattering processes. The present study focused on the the sound velocity which is determined by the density and the elastic constant of a solid. The sound velocity has three polarizations, including one longitudinal mode and two transverse modes, as shown in Figure 10.

A pulse-echo method was used to measure these sound velocities with a 30 MHz longitudinal transducer and a 20 MHz transverse transducer. The measured values are listed in Table 1. The corresponding values for TiS_2_ are also included for comparison.

**Table 1:** Densities, longitudinal and transverse sound velocities, and shear moduli of TiS_2_, (BiS)_1.2_(TiS_2_)_2_, (SnS)_1.2_(TiS_2_)_2_ and (PbS)_1.18_(TiS_2_)_2_.

Material	*ρ* g/cm^3^	*V*_L_ m/s	*V*_T1_ m/s	*V*_T2_ m/s	*G*_1_ GPa	*G*_2_ GPa
TiS_2_	3.21	5284	2799	3295	25.0	34.7
(BiS)_1.2_(TiS_2_)_2_	4.57	3662	1350	1688	8.3	13.0
(PbS)_1.18_(TiS_2_)_2_	4.69	3834	1120	1837	5.9	15.8
(SnS)_1.2_(TiS_2_)_2_	3.87	4111	1578	2352	9.6	21.4

Compared with pure TiS_2_, the longitudinal velocities of the misfit layer compounds are a little decreased, which can be attributed to the increase of density. In contrast, the transverse sound velocities, especially *V*_T1_, apparently decreased, which arises from the softening of atomic bonding. The transverse polarization is a kind of shear movement, and the velocity is determined by shear modulus as follows:

(6) $$V_{T}=\sqrt{\frac{G}{\rho }}$$

where *G* is the shear modulus and *ρ* is the density. The shear modulus is calculated by the above equation and shown in Table 1. The shear moduli of the misfit layer compounds are much lower than those of pure TiS_2_ due to the intercalation of the *M*S layers into the TiS_2_ layers. It can also be seen that the velocities of the two transverse waves (*V*_T1_ and *V*_T2_) are different, as *V*_T1_ is mainly determined by the interlayer bonding while *V*_T2_ is determined by the intralayer bonding. For *V*_T1_, the weak interlayer bonding between the *M*S layer and TiS_2_ layer arises either from the electrostatic interaction due to electron transfer between these layers or a weak covalent force between the *M* atom and the sulfur atoms in the TiS_2_ layers [14,15]. For *V*_T2_, the intralayer bonding is weakened, possibly due to the incommensurate structure or disruption of periodicity of TiS_2_ layers in the direction perpendicular to the layers by the intercalated *M*S layers.

It has been shown that the sound velocity decreased in the misfit layer compounds due to the weakened bonding, which can partially account for their low thermal conductivity. However, further investigation is required to understand the compositional dependence of lattice thermal conductivity of the (*M*S)_1+*x*_(TiS_2_)_2_ compounds, which mainly differ in phonon mean free path. It is anticipated that the electron transfer may play a role in determining the phonon transport, because (BiS)_1.2_(TiS_2_)_2_ which has the most electron transfer exhibits the lowest lattice thermal conductivity.

### 2.4. ZT value

**Figure 11:**
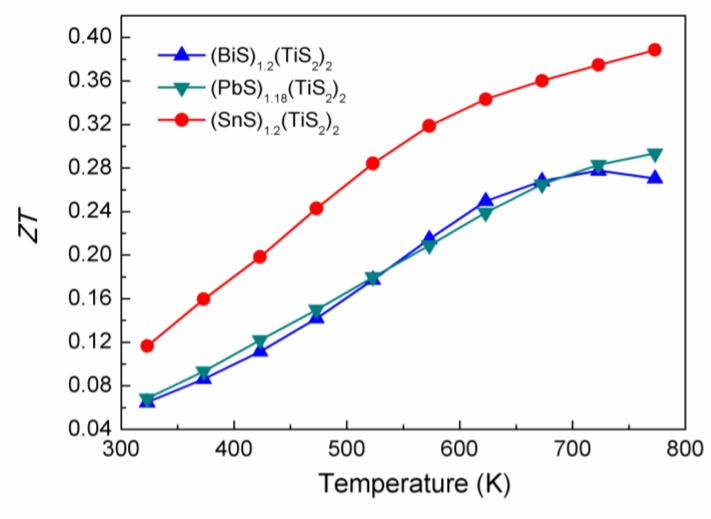
*ZT* values of (BiS)_1.2_(TiS_2_)_2_, (SnS)_1.2_(TiS_2_)_2_ and (PbS)_1.18_(TiS_2_)_2_.

The *ZT* values of the three misfit layer compounds are shown in Figure 11. These misfit layer compounds show an intermediate *ZT* value of 0.28~0.37 at 700 K. The (SnS)_1.2_(TiS_2_)_2_ compound shows the highest *ZT* value among the three investigated composition and can be considered as promising medium-temperature *n*-type thermoelectric materials, as it is composed of non-toxic, non-hazardous and naturally abundant elements. Since these misfit layer compounds have extremely low thermal conductivity and rather high carrier concentration, the reduction in carrier concentration can reduce the electronic thermal conductivity and optimize the power factor simultaneously, and much higher *ZT* value can be expected to be achieved.

## 3. Experimental Section

The (*M*S)_1+*x*_(TiS_2_)_2_ (*M* = Bi, Sn, Pb) powders were prepared using a solid-liquid-vapor reaction method [16,17]. For each composition, the *M*, S, Ti powders were mixed in the molar ratio of 1:2:5 and then sealed in an evacuated silica tube. The silica tube was then fired in an electric furnace at 500 °C for 12 h, then at 800 °C for 48 h and finally cooled down to room temperature. The obtained powders with luster were ground and sieved. The spark plasma sintering (SPS) method (SPS-1050, Sumitomo Mining Coal Mining Co. Ltd.) was used to densify the powders at 700 °C for 10 min under the pressure of 50 MPa into a pellet with diameter of 15 mm and thickness of 6 mm. Since the microstructure of the pellet is highly anisotropic, it was deliberately machined for thermoelectric properties measurements in the direction perpendicular to pressure.

The densities of the samples were measured using the Archimedes method. The phase composition was characterized by the X-ray diffraction measurements (RINT-2100, Rigaku). The Seebeck coefficient and electrical conductivity were measured simultaneously by a conventional steady state method and a four-probe method, respectively, in an Ar atmosphere at 300–773 K (RZ-2001K, Ozawa Science). The carrier concentration was determined by Hall effect measurement with a van der Pauw electrode configuration under vacuum of 10^−3^ Pa over the same temperature range (Resi Test 8300, Toyo Technica). The heat capacity and the thermal diffusivity were measured by differential scanning calorimetry (DSC-2910, TA Instruments) and laser-flash method (TC-9000V, ULVAC-RIKO), respectively. The thermal conductivity was calculated as a product of density, heat capacity and thermal diffusivity. The sound velocities including one longitudinal and two transverse modes were measured by the ultrasonic pulse-echo method (Model 5800 PR, Olympus).

## 4. Conclusions

Thermoelectric properties of a series of misfit layer compounds (*M*S)_1+*x*_(TiS_2_)_2_ (*M* = Pb, Bi, Sn) are studied. These compounds appear to be promising for medium-temperature *n*-type thermoelectric materials. This naturally modulated structure shows low lattice thermal conductivity close to or even lower than the predicted minimum thermal conductivity. Measurement of sound velocities shows that the ultra-low thermal conductivity partially originates from the softening of the transverse modes of lattice wave due to weak interlayer bonding. Meanwhile, electron transfer from the *M*S layer to the TiS_2_ layer deteriorates the thermoelectric performance by reducing the power factor and increasing the electronic thermal conductivity. The SnS intercalation compound (SnS)_1.2_(TiS_2_)_2_ has the least electron transfer and the *ZT* value reaches 0.37 at 700K. Reduction in the carrier concentration in these misfit layer compounds is required to achieve higher *ZT* value.
